# Supplementary material for: Moiré magnetism in CrBr3 multilayers emerging from differential strain
Source: Nat Commun. 2024 Nov 29;15:10377. doi: 10.1038/s41467-024-54870-2 (PMC11606980; doi:10.1038/s41467-024-54870-2)
Supplement: Supplementary file 1 — Supplementary Information [file 41467_2024_54870_MOESM1_ESM.pdf]

Supplementary Information for

Moiré magnetism in $CrBr_3$ multilayers emerging from differential strain

Fengrui Yao[1,2*], Dario Rossi[3], Ivo A. Gabrovski[1], Volodymyr Multian[1,2,4], Nelson Hua[5], Kenji Watanabe[6], Takashi Taniguchi[7], Marco Gibertini[8,9], Ignacio Gutiérrez-Lezama[1,2], Louk Rademaker[1*], and Alberto F. Morpurgo[1,2*]

[1]*Department of Quantum Matter Physics, University of Geneva, 24 Quai Ernest Ansermet, CH-1211 Geneva, Switzerland*

[2]*Group of Applied Physics, University of Geneva, 24 Quai Ernest Ansermet, CH-1211 Geneva, Switzerland*

[3]*Department of Theoretical Physics, University of Geneva, 24 Quai Ernest Ansermet, CH-1211 Geneva, Switzerland*

[4]*Advanced Materials Nonlinear Optical Diagnostics lab, Institute of Physics, NAS of Ukraine, 46 Nauky pr., 03028 Kyiv, Ukraine*

[5]*Laboratory for X-ray Nanoscience and Technologies, Paul Scherrer Institut, CH-5232 Villigen PSI, Switzerland*

[6]*Research Center for Electronic and Optical Materials, National Institute for Materials Science, 1-1 Namiki, Tsukuba, 305-0044, Japan*

[7]*Research Center for Materials Nanoarchitectonics, National Institute for Materials Science, 1-1 Namiki, Tsukuba, 305-0044, Japan*

[8]*Dipartimento di Scienze Fisiche, Informatiche e Matematiche, University of Modena and Reggio Emilia, IT-41125, Modena, Italy*

[9]*Centro S3, CNR-Istituto Nanoscienze, IT-41125, Modena, Italy*

*Correspondence: fengrui.yao@unige.ch; louk.rademaker@unige.ch; alberto.morpurgo@unige.ch

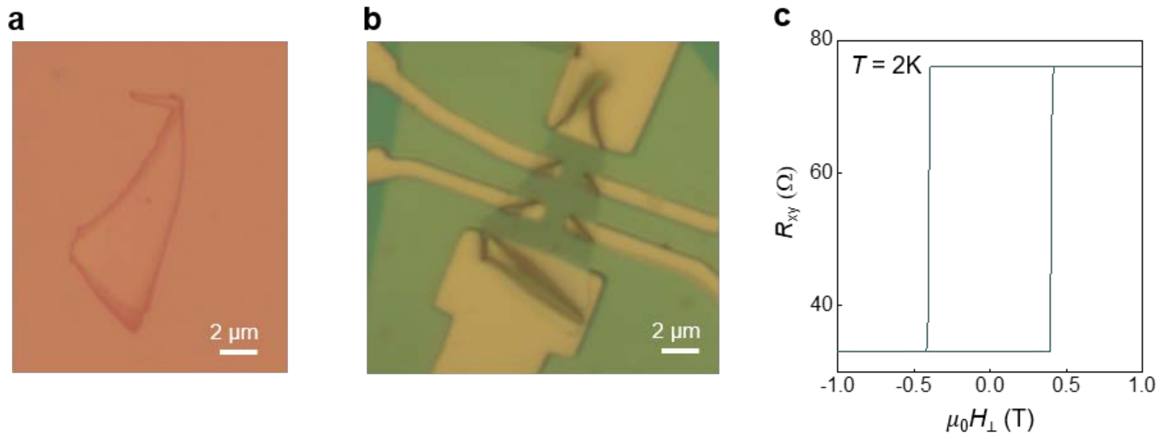

Supplementary Fig. 1. Transport characterization of thin Fe_3GeTe_2 (FGT) flakes. **a**, Optical microscopy image of a thin FGT crystal (11 nm) exfoliated on a 285 nm SiO_2/Si substrate. **b,** Optical image of a Hall bar device realized using the crystal shown in (**a**), with the FGT crystal fully encapsulated in between h-BN exfoliated layers. c, Longitudinal resistance (R_{xy}) of the FGT Hall bar device measured at $T = 2$ K, showing anomalous Hall effect. From these measurements we determine that the switching field of the material is 400 mT at 2 K, consistent with the switching field observed in the FGT/$CrBr_3$/Graphene (Gr) tunnel barrier devices discussed in the main text (see Fig. 2f and Fig. 3d)

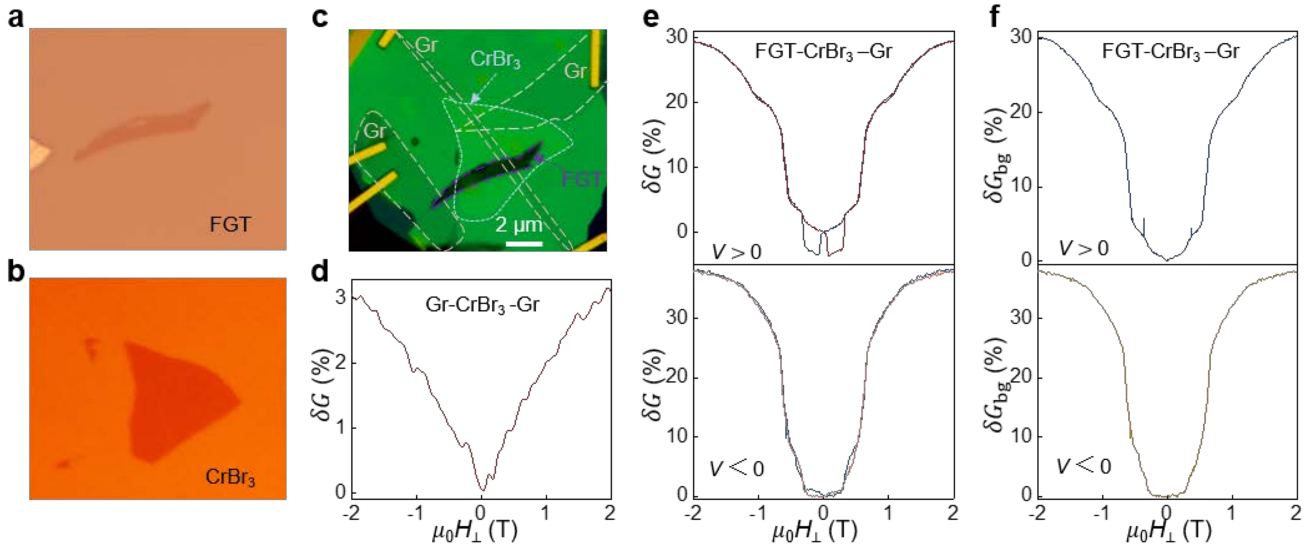

Supplementary Fig. 2: Magnetoconductance of a second device with nearby FGT/$CrBr_3$/Gr and Gr/ $CrBr_3$/Gr junctions (all data taken at $T = 2$ K). Optical microscopy image of (**a**) the FGT crystal used as an electrode, and (**b**) of the $CrBr_3$ used as the barrier, as exfoliated onto a 285 nm SiO_2/Si substrate**. c**, Optical microscope image of the assembled device containing an FGT/$CrBr_3$/Gr and a Gr/$CrBr_3$/Gr junction, realized on the same $CrBr_3$ multilayer (around 4 nm thick). **d,** The tunneling magnetoconductance (δG) of the Gr/$CrBr_3$/Gr barrier is small, confirming that the $CrBr_3$ multilayer is fully ferromagnetic (compare with Fig. 1 g,h). Tunneling magnetoconductance (**e**) and extracted magnetoconductance background (**f**) of the FGT/$CrBr_3$/Gr junction, with electrons injected from the FGT ($V > 0$, top panel) and the Gr ($V < 0$, bottom panel) electrode. The behavior observed is qualitatively identical to that of the device discussed in the main text (Fig. 3) and confirms that an FGT electrode induces antiferro-magnetic regions in the $CrBr_3$ layers underneath, which are otherwise ferromagnetic in their pristine form.

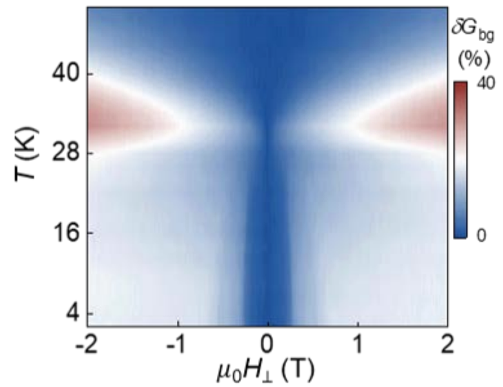

Supplementary Fig. 3. Color plot of the temperature-dependent magnetoconductance measured on a Gr/twist CrBr3 (θ = 2 °)/Gr junction. When plotted as a function of magnetic field and temperature, the magnetoconductance of Gr/twist CrBr3/Gr devices is virtually identical to that measured in FGT/CrBr3/Gr junctions (see Fig. 3f in the main text). The data show the coexistence of ferromagnetism and antiferromagnetism, as the 'lobes' near *T*c originate from the presence of ferromagnetism, and the temperature evolution of the magnetoconductance background (all features shift to lower fields as the temperature increases and disappear as *T* approaches *T*C) originates from the presence of antiferromagnetism.

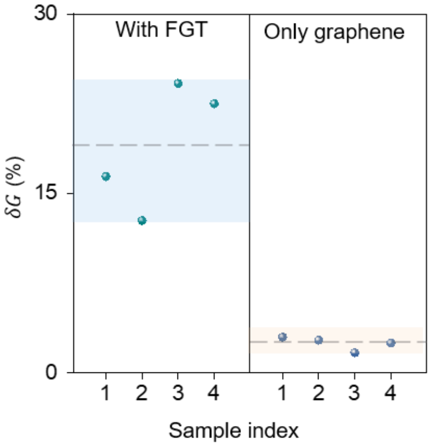

Supplementary Fig. 4. Magnetoconductance *δG* (*H* = 1 T, 2 K) of CrBr3 barriers with and without FGT contacts. Left: magnitude of the magnetoconductance background (average of the two values measured at opposite biases) for the four devices investigated having a FGT contact. Right: magnitude of the magnetoconductance of four Gr/ CrBr3/Gr devices measured at H = 1 T and T = 2K. The horizontal dashed lines in the two panels represent the respective average value. From this analysis we conclude that the average magnetoconductance of FGT/CrBr3/Gr junctions is around 22%, one order of magnitude larger than that of Gr/ CrBr3/Gr junctions, which is 2%. In all cases, the CrBr3 multilayer employed to realize the devices is AB-stacked (i.e., ferromagnetic in its pristine form).

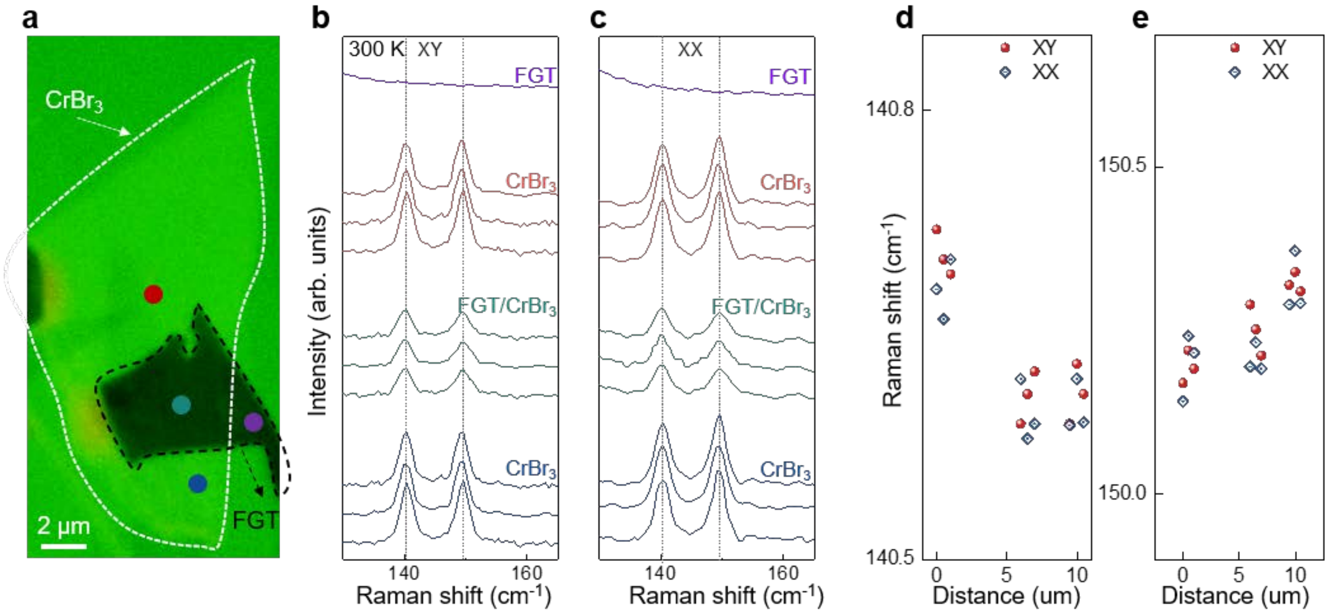

Supplementary Fig. 5. Comparison of room temperature $CrBr_3$ Raman spectra next to and under an FGT crystal (data measured at T = 300 K). a, Optical microscopy image of the tunnel junction after assembly of the van der Waals heterostructure (FGT/$CrBr_3$/Gr and Gr/$CrBr_3$/Gr junctions encapsulated by h-BN). The colored dots mark the positions where the Raman spectrum was measured. **b,c,** Raman spectra, measured at different locations – under crossed (XY, **b**) and parallel (XX, **c**) polarization configurations of the incident and detected light. The purple curves, measured on the FGT electrode (around the purple dot in panel **a**), show no prominent peaks in the studied wavelength range. The red and blue curves are the Raman spectra, measured at positions next to the FGT electrode (three different positions around the red and blue dots in panel **a**). The green curves are measured at different positions under the FGT electrode (three different positions around the green dot in panel **a**). In contrast to what happens at T = 20 K (see Fig. 4), at room temperature, the peak positions measured in $CrBr_3$ under an FGT crystal are the same as the peak positions measured in $CrBr_3$ away from the FGT crystal**. d,e,** Extracted peak position of the two individual modes as a function of position on the $CrBr_3$ multilayer where the measurements are done (on the x-axis, Distance = 0 μm corresponds to the position of the red dot in panel **a**; the FGT crystal is located at Distances between approximately 3 μm and 5μm). No difference depending on the polarization configuration is observed at room temperature.

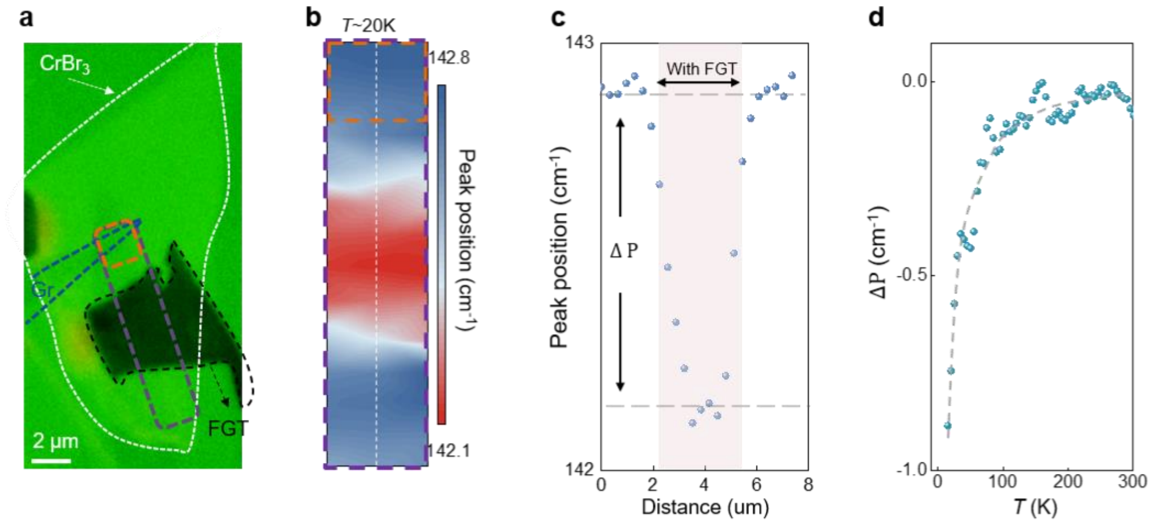

Supplementary Fig. 6. Spatial Raman Mapping of CrBr3 under FGT and under graphene. a, Optical image of a van der Waals heterostructure that contains regions where a CrBr3 multilayer (outlined by the white dotted line) is covered by a FGT multilayer (outlined by the black dashed line) and by a graphene strip (outlined by a dark blue dotted line). The rectangle delimited by the dashed purple line corresponds to the area over which the Raman signal has been mapped. The position of the Eg mode near 140 cm^{-1} extracted from these measurements is shown in panel **b.** The mapping indicates a rather uniform peak shift in the FGT-covered region, while no shift is observed in the graphene-covered CrBr3 region (located inside the rectangle delimited by the orange line). No evidence of multiple domains with varying stacking configurations can be seen in the whole map. Measurements were conducted under parallel (XY) polarization at ~20 K. **c,** Line scan of Raman peak positions extracted from the spatial mapping in panel **b**, taken along the white dashed line, quantifying the peak shift (ΔP) in the FGT-covered CrBr3 region. **d,** Shift of the position (ΔP) of the Eg mode near 140 cm^{-1} as a function of temperature (T). The shift is gradual, becomes large below 100 K, and exhibits no sharp features originating from structural or magnetic phase transitions (the dashed line is a guide to the eye).

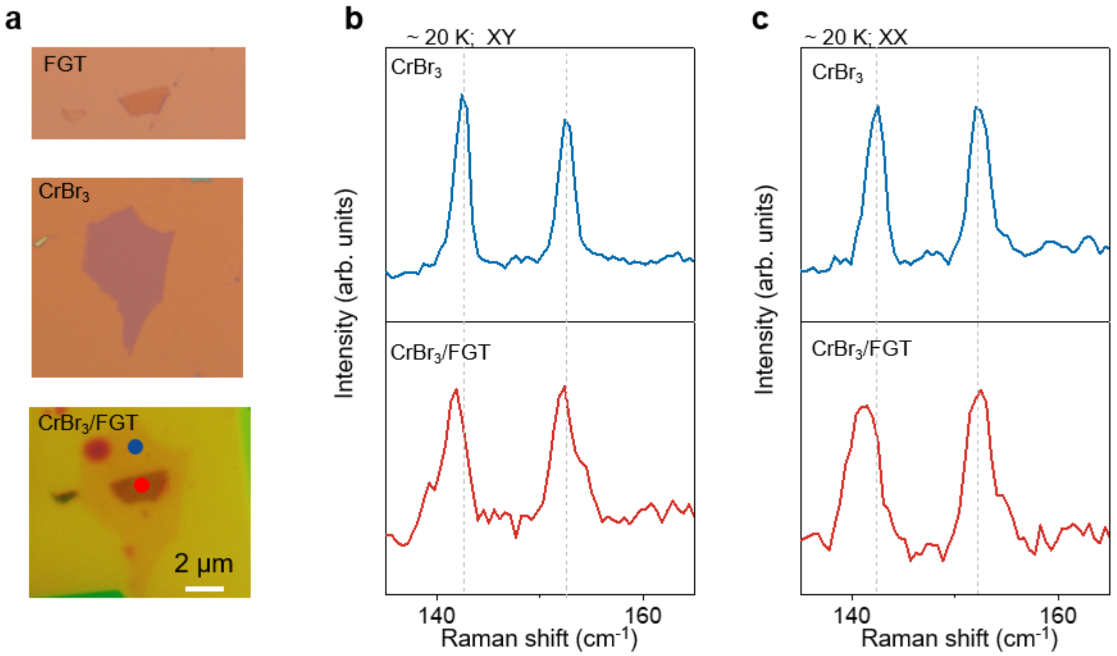

Supplementary Fig. 7. Raman spectra of CrBr3 a FGT multilayer. a, Optical microscope images of a FGT (top panel) and of a CrBr3 (middle panel) multilayer that we employ to assemble a CrBr3/FGT heterostructure (encapsulated in between h-BN crystals) with CrBr3 on top of FGT (in all devices employed to measure transport, FGT was placed on top of CrBr3). The blue and red colored dots indicate the positions where Raman spectra were measured. **b, c,** Raman spectra obtained at different positions on the heterostructure under crossed (XY, **b**) and parallel (XX, **c**) polarization configurations. Blue curves represent measurements taken on CrBr3 next to the FGT electrode (at the position indicated by the blue dot in panel **a**); red curves correspond to data measured on top of the CrBr3/FGT heterostructure (at the position indicated by the red dot in panel **a**). The position of the peak shifts (and the peak broadens, as expected in the presence of two split peaks) when measured on CrBr3 on FGT, as compared to the measurement done on CrBr3 next to the FGT. The effect is identical to the one seen when FGT is placed on top of CrBr3 (shown in Fig. 5), indicating that the strain that FGT induces in CrBr3 is approximately the same in the two cases, irrespective of what material is on top.

2 Theoretical model for the magnetization

Transport measurements are notoriously difficult to theoretically model quantitatively. However, as mentioned in the main text, a positive tunneling magnetoconductance through a $CrBr_3$ barrier originates from the lowering of the barrier height due to the alignment of spins in $CrBr_3$. It follows that the magnetoconductance correlates directly to the barrier magnetization and, indeed, in ferromagnetic $CrBr_3$ barriers (i.e., AB-stacked $CrBr_3$) it has been shown that the magnetoconductance is an approximately parabolic function of magnetization[13], $\delta G \sim (\delta M)^2$. For these reasons, we expect that the magnetoconductance in strained $CrBr_3$ barriers should also be directly related to their magnetization. Unlike the magnetoconductance, the magnetization is much simpler to model and study analytically and numerically. We therefore construct a spin model in a magnetic field to model the $CrBr_3$ multilayer, and compare the resulting magnetization at different applied magnetic fields to the magnetoconductance.

2.1 General model

$CrBr_3$ is considered to have a Heisenberg interaction (with negligibly small anisotropy) and a non-negligible single-ion easy-axis (out-of-plane) anisotropy (SIA)[10]. We therefore write the spin Hamiltonian for a layer l of $CrBr_3$ as a Heisenberg model with SIA,

$$\mathcal{H}_l = \frac{1}{2}\sum_n \sum_{\boldsymbol{\delta}} \tilde{J}_{\boldsymbol{\delta}}\, \boldsymbol{S}_l(\boldsymbol{x}_n)\cdot \boldsymbol{S}_l(\boldsymbol{x}_n+\boldsymbol{\delta}) - \tilde{h}\sum_n S_l^z(\boldsymbol{x}_n) - \tilde{d}\sum_n \left(S_l^z(\boldsymbol{x}_n)\right)^2 \tag{1}$$

where $J_{\boldsymbol{\delta}}$ is the Heisenberg exchange between two sites connected by a lattice vector $\boldsymbol{\delta}$, $\boldsymbol{x}_n$ is the position of lattice site n, $\tilde{h}$ is an external out-of-plane magnetic field parameter, and $\tilde{d}$ is the anisotropy. As stated below in Sec. 2.2, we expect a difference in strain to be present between neighboring layers, which leads to a moiré pattern. $CrBr_3$ has been shown to exhibit a stacking-dependent interlayer Heisenberg exchange[4], which results in a position-dependent interlayer exchange interaction in our model. We write the interlayer Hamiltonian as

$$\mathcal{H}_{l,\perp} = \sum_n \tilde{J}_\perp(\boldsymbol{x}_n)\, \boldsymbol{S}_l(\boldsymbol{x}_n)\cdot \boldsymbol{S}_{l+1}(\boldsymbol{x}'_n) \tag{2}$$

where $\boldsymbol{x}'_n$ is the position of the lattice site in layer $l+1$ that is closest to $\boldsymbol{x}_n$. The total Hamiltonian for L layers is then

$$\mathcal{H} = \sum_l^L \mathcal{H}_l + \sum_l^{L-1} \mathcal{H}_{l,\perp}\,. \tag{3}$$

If any sort of noncollinear magnetism is present in the system, the spin orientation will vary on the moiré scale, which is much larger than the atomic scale. This elicits rewriting the Hamiltonian in the continuum limit by taking the substitutions

$$\begin{aligned} \sum_n f(\boldsymbol{x}_n) \quad &\longrightarrow \quad 2\,V_u^{-1}\int d^2x\, f(\boldsymbol{x}) \\ \boldsymbol{S}_l(\boldsymbol{x}_n) \quad &\longrightarrow \quad S\,\boldsymbol{m}_l(\boldsymbol{x}) \end{aligned}$$

and expanding the Heisenberg exchange term in powers of $\boldsymbol{\delta}$. Here, V_u is the unit cell area, $S=\frac{3}{2}$ is the total on-site spin, $\boldsymbol{m}$ is the magnetization unit vector, and the factor 2 accounts for the presence of two spins per unit cell in hexagonal $CrBr_3$. Therefore, we write the Hamiltonian density for a two-layer system as

$$\mathscr{H}(\boldsymbol{x}) = \sum_l^2 \frac{\rho^{ab}}{2}\left(\frac{\partial \boldsymbol{m}_l}{\partial x_a}\right)\cdot\left(\frac{\partial \boldsymbol{m}_l}{\partial x_b}\right) - d\,(m_l^z)^2 - h m_l^z + J_\perp(\boldsymbol{x})\,\boldsymbol{m}_1\cdot\boldsymbol{m}_2 \tag{4}$$

where $\rho^{ab} = -S^2\,V_u^{-1}\sum_{\boldsymbol{\delta}} \tilde{J}_{\boldsymbol{\delta}}\delta^a\delta^b$ is the spin stiffness tensor, $d = 2\,\tilde{d}\,S^2\,V_u^{-1}$, $J_\perp = 2\,\tilde{J}_\perp\,S^2\,V_u^{-1}$ and $h = 2\,\tilde{h}\,S\,V_u^{-1}$. In a honeycomb lattice, the spin stiffness tensor is diagonal. We observe that if any noncollinear texture exists due to a sufficiently strong interlayer coupling $J_\perp$, then the magnetization will be coplanar; because noncoplanar features (such as skyrmions) will cost additional energy. Having only coplanar order, we parametrize the magnetization as $\boldsymbol{m}_l(\boldsymbol{x}) = \hat{\boldsymbol{x}}\sin\varphi_l(\boldsymbol{x}) + \hat{\boldsymbol{z}}\cos\varphi_l(\boldsymbol{x})$, and write the Hamiltonian density in terms of the polar angles as

$$\mathscr{H}(\boldsymbol{x}) = \frac{\rho}{2}\left((\nabla\varphi_1)^2 + (\nabla\varphi_2)^2\right) + J_\perp(\boldsymbol{x})\cos(\varphi_1-\varphi_2) - d\left(\cos^2\varphi_1 + \cos^2\varphi_2\right) - h\left(\cos\varphi_1+\cos\varphi_2\right)\,. \tag{5}$$

The energy minima must satisfy the Euler-Lagrange equations

$$\begin{aligned} \rho\nabla^2\varphi_1 &= d\sin 2\varphi_1 + h\sin\varphi_1 - J_\perp(\boldsymbol{x})\sin(\varphi_1-\varphi_2) \\ \rho\nabla^2\varphi_2 &= d\sin 2\varphi_2 + h\sin\varphi_2 + J_\perp(\boldsymbol{x})\sin(\varphi_1-\varphi_2)\,. \end{aligned} \tag{6}$$

Source		$\tilde{J}_{\text{NN}}$	$\tilde{J}_\perp$ (AA)	$\tilde{d}$
First-principle calculations	Cai[2]	1.36	-	0.04
	Akram[1]	3.42	-	0.03
	Tong[12]	1.5	-	0.026 (exp.)
	Gibertini[4]	-	0.62	-
	Sun[11]	0.77-0.98	-	0.012
	Singh[10]	1.9	-	0.08
Experimental fitting	Fumega (+DFT)[3]	2.5	0.5	-
	Nikitin[6]	1.485	-	0.028
	Cai[2]	1.48	-	0.02

Supplementary Table 1: Comparison of ferromagnetic nearest-neighbor Heisenberg exchange $\tilde{J}_{\text{NN}}$ and single-ion easy-axis anisotropy $\tilde{d}$ from different sources in the literature. All values are in meV.

Alternatively, the Hamiltonian density and the corresponding Euler-Lagrange equations can be written in terms of the (anti)symmetric polar angles $\varphi_a = \varphi_1 - \varphi_2$ and $\varphi_s = \varphi_1 + \varphi_2$ like

$$\begin{aligned} \mathscr{H}(\boldsymbol{x}) &= \frac{\rho}{4}\big((\nabla\varphi_s)^2 + (\nabla\varphi_a)^2\big) + \cos\varphi_a\big(J_\perp(\boldsymbol{x}) - d\cos\varphi_s\big) - 2h\cos\frac{\varphi_s}{2}\cos\frac{\varphi_a}{2} \\ \frac{\rho}{2}\nabla^2\varphi_s &= d\cos\varphi_a\sin\varphi_s + h\cos\frac{\varphi_a}{2}\sin\frac{\varphi_s}{2} \\ \frac{\rho}{2}\nabla^2\varphi_a &= -\sin\varphi_a\big(J_\perp(\boldsymbol{x}) - d\cos\varphi_s\big) + h\sin\frac{\varphi_a}{2}\cos\frac{\varphi_s}{2}\,, \end{aligned} \tag{7}$$

which is used by Ref. 5 and is, in general, easier to work with analytically.

In principle, a Dzyaloshinskii-Moriya interaction, which has been reported between next-nearest-neighbors in $CrBr_3$[2], can stabilize noncoplanar magnetization. However, we find that due to the crystal symmetries of $CrBr_3$, the DM interaction energy cancels to zero in the lowest few orders of the continuum expansion. We conclude that even though the presence of an in-plane DM interaction may produce topological magnetic excitations at the edges of the Brillouin zone[7,8,9], DM interactions can be neglected when looking for the magnetization ground state.

2.2 $CrBr_3$ parameters

The parameters used in our theoretical spin model have been studied experimentally and numerically in the past. Table 1 summarizes some of the results. Setting the lattice constant to 1, we have $V_u = \sqrt{3}/2$ and this allows us to convert these values to the continuum model parameters – for example $\rho = 1.61$ meV, $d = 0.2$ meV using Ref. 2. As Table 1 suggests, however, there is no agreement on the precise values for the magnetic parameters in $CrBr_3$, but they do provide an estimate for the order of magnitude of the parameters. We, therefore, vary the stiffness and anisotropy in our numerical calculations.

To estimate the interlayer coupling, we combine the ab initio results of Ref. 4 with experimental results on $CrBr_3$ multilayers. Ref. 14 studied the response of different antiferromagnetic stackings to an applied in- and out-of-plane magnetic field. In particular, there is a critical orthogonal field at which two neighboring layers align, which is 0.55 T for the M metastable stacking and 0.2 T for the AA metastable stacking. These critical spin-flip fields are directly proportional to the interlayer antiferromagnetic Heisenberg coupling strength. For this reason, we re-scale the ab initio interlayer Heisenberg coupling to have the correct ratio of $\tilde{J}_\perp$ in the M'- and AA-stacked regions. In the rescaling process, we preserve the maximum and minimum values of the original data. The result is presented in Supplementary Figure 8. In particular, we notice that the interlayer Heisenberg coupling at and near the AA antiferromagnetic region has a reduced magnitude after the rescaling.

Localized moiré interface

In this work we argue that the $CrBr_3$ multilayer experiences differential strain due to its coupling to the FGT electrode. The strong metallic coupling of FGT to $CrBr_3$ ensures that the top layer of $CrBr_3$ experiences a nonzero strain $\epsilon^{(1)}$. The question is how and whether this strain will propagate through the other layers, each of which can be characterized by a strain parameter $\epsilon^{(\ell)}$.

In the simplest approximation, there are now three energy contributions that affect the strain propagation:

- The **in-plane strain energy** increases quadratically with the effective strain of each layer ℓ, giving $U \sim \sum_\ell (\epsilon^{(\ell)})^2$. This energy therefore favors as few layers as possible to have nonzero strain.
- The **stacking energy** between adjacent layers is minimized for perfect AB stacking. However, when two adjacent layers have different strain, $\epsilon^{(\ell)} \neq \epsilon^{(\ell+1)}$, a large-scale moiré structure arises with locally different stacking. The stacking energy is therefore minimized when neighboring layers have the same strain.

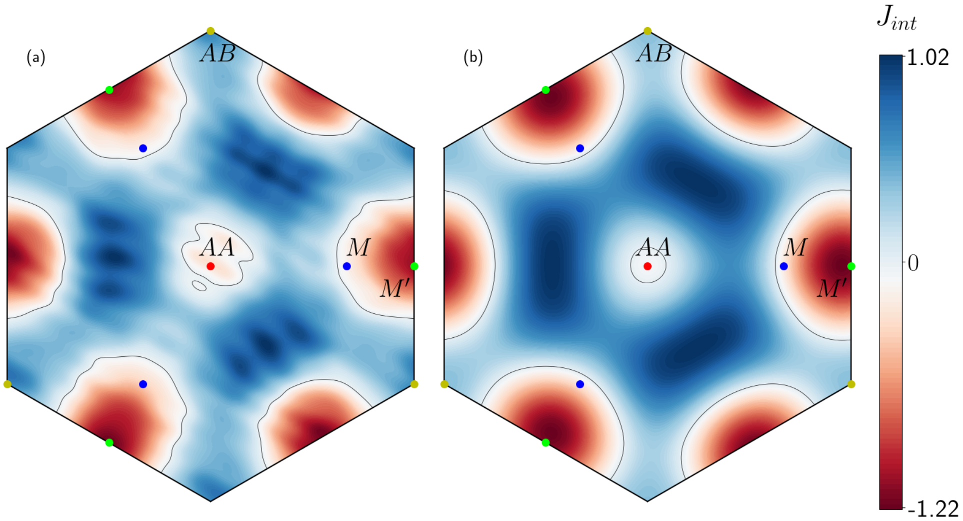

Supplementary Figure 8: **Interlayer exchange interaction calculated from first principles** (**a.**) and rescaled (**b.**) to be consistent with the relative switch fields in AA and M stackings. High symmetry points are highlighted: AB, AA, M and M'.

- The **magnetic energy** [see previous section] is minimized when the interlayer coupling is ferromagnetic. Any non-collinear texture, which is inevitable when there is a moiré interface, costs energy.

These three contributions together imply, without having to calculate the precise balance, that the total energy is minimized with having a *single moiré interface* where the $CrBr_3$ in-plane lattice constant goes from the FGT-mandated value to the relaxed monolayer value. Where precisely this interface occurs in the multilayer depends on the interplay between the magnetic energy, the in-plane strain energy and the possible effect of lattice relaxation. The experimental evidence that ferromagnetism and antiferromagnetism coexist in the $CrBr_3$ multilayer further supports the notion that a moiré interface appears. We conclude that we can model the magnetic texture of the multilayer through the simple bilayer model presented in the previous section.

Note that since all layers on each side of the moiré interface have the same magnetic texture, the effective spin stiffness of at the interface increases with the multilayer thickness.

Magnitude of strain

Since both FGT and $CrBr_3$ have hexagonal unit cells with a lattice constant ratio between 1.5 and 1.6, we expect that a strong binding between FGT and $CrBr_3$ will force the more elastic layer ($CrBr_3$) to strain in order to achieve some commensurability. Depending on the angle at which FGT and $CrBr_3$ make contact, the strain required to achieve commensurability can vary a lot and it can be different along the two lattice vectors. Based on a simple geometric matching of the lattice constants with varying twist angles, we find that realistic strains of the top $CrBr_3$ layer can vary between approximately 0.3% and 3%.

2.3 Without a magnetic field

In the absence of a magnetic field, a perturbative analytical solution for the bilayer system can be found in Ref. 5 in the case when $\int_{\text{moiré}} d^2x J_\perp(\boldsymbol{x}) = 0$. Depending on the strain, spin stiffness, and anisotropy, they find three ground states:

- Collinear (c) – All spins in the two layers are oriented out-of-plane. Characterized by strong anisotropy.
- Twisted-s (*tw-s*) – $\varphi_s = 0/\pi$ and φ_a follows the interlayer coupling. Characterized by weak anisotropy.
- Twisted-a (*tw-a*) – One layer is pinned $\varphi_1 = 0$ and the other aligns parallel or anti-parallel, depending on the local value of $J_\perp$. Characterized by strong interlayer coupling.

In the case of $CrBr_3$, regardless of the precise values, the interlayer Heisenberg coupling is always at least one order of magnitude larger than the anisotropy. This leads us to believe that at realistic strains, which produce very large moiré unit cells, the ground state magnetisation is in a *tw-a* phase. The transitions from *tw-a* to c and from *tw-a* to *tw-s* have a jump in the magnetization and resemble a spin flip and a spin flop transition, respectively. The transition from *tw-s* to c is continuous in the magnetization. One might expect that applying a magnetic field can drive these transitions.

2.4 Adding a magnetic field

In the presence of a magnetic field, the model becomes impossible to solve analytically. Intuitively, we expect that in the *tw-a* phase, applying a magnetic field will move the domain wall; in the *tw-s* phase, we expect the magnetic field to cant all spins towards the direction of the field, thus, also moving the domain wall. In the following section, we give additional motivation as to why this occurs.

2.4.1 Domain wall movement in the *tw-a* phase

In a moiré bilayer, the interlayer coupling $J_\perp$ in $CrBr_3$ has domes of antiferromagnetic exchange on a ferromagnetic background. To understand the behavior of the domain wall in the *tw-a* phase, let us consider a simple model where one circular antiferromagnetic dome with radius $r_\perp$ sits at the origin. We write the interlayer Heisenberg coupling as $J_\perp(r - r_\perp)$, which goes from positive to negative at $r = r_\perp$. One layer is pinned, which leaves us with only one degree of freedom per spin – the polar angle φ of the unpinned layer. Working in radial coordinates, we can rewrite the Hamiltonian density as

$$\mathscr{H} = \frac{\rho}{2}\left(\frac{\partial\varphi}{\partial r}\right)^2 - d\cos^2\varphi + \big(J_\perp(r - r_\perp) - h\big)\cos\varphi\,.$$

In the absence of a magnetic field and interlayer coupling, the domain wall is an excited state topological defect. We can solve the Euler-Lagrange equations of the Hamiltonian density to give a solution for the domain wall

$$\cos\varphi = \tanh\left(\frac{r - r_0}{w}\right) \tag{8}$$

where $w = \sqrt{\rho/(2d)}$ is the effective width of the domain wall and r_0 is an integration constant, which gives the position of the domain wall. In $CrBr_3$, with the parameters given above, we can estimate that the domain wall width will have a lower boundary of 2-3 lattice constants, which is very small compared to the moiré length. However, in a thicker layer where the effective spin stiffness is increased, the domain wall will be larger. Using the domain wall solution as an ansatz for the full Hamiltonian density, we can find the position of the domain wall by minimizing the energy $\frac{\partial}{\partial r_0}\int_0^\infty dr\, r\,\mathscr{H} = 0$. This results in an integral equation for the position of the domain wall

$$2dw\left[1 + \tanh\left(\frac{r_0}{w}\right)\right] = -\int_0^\infty dr\,\frac{r}{w}\big(J_\perp(r - r_\perp) - h\big)\,\mathrm{sech}^2\left(\frac{r - r_0}{w}\right)\,. \tag{9}$$

In the limit $w \ll r_0$, we can approximate the equation like $2dw = -r_0\big(J_\perp(r_0 - r_\perp) - h\big)$. This tells us that when anisotropy is small and there is no magnetic field, the domain wall position is fixed at $r_0 = r_\perp$. Applying a magnetic field shifts the domain wall towards the origin and increases the net magnetization.

2.4.2 Spin canting in the *tw-s* phase

Similarly to an antiferromagnet undergoing a spin-flop transition, we expect that after a transition from a *tw-a* phase to a *tw-s* phase, the spins will cant out-of-plane when the magnetic field is further increased. This can be quantified by considering a small magnetic field perturbation to the solution presented in Ref. 5.

Therefore, an antiferromagnetic dome, starting in the *tw-a* phase, shrinks upon application of an out-of-plane magnetic field. There are two possible paths that the system can then take:

1. A sharp transition to a *tw-s*, phase, which then continuously transforms into a *c* phase at larger fields.
2. A sharp transition to a *c* phase.

Whether or not the system passes through the *tw-s* phase depends on the spin stiffness and anisotropy, as well as the shape and magnitude of the interlayer coupling at the dome (e.g. if the interlayer coupling potential varies very smoothly, the *tw-a* domain wall might shrink all the way to a single spin before flipping and never transitioning to a *tw-s* phase). This also means that the domes, corresponding to local AA and M stacking, may have different transitions once a magnetic field is applied. A numerical solution is required to find out what exactly happens to the magnetization in a $CrBr_3$ bilayer.

3 Solving the model numerically

We solve the model numerically with a gradient descent routine. We define the spins of one moiré unit cell on a grid of linear dimension D. To compute the energy Equation 5, we need to evaluate the gradient of the phases (polar angles) $\varphi_{1/2}$, and to compute the functional derivatives Equation 6, we need to evaluate the Laplacian. Since the phases are defined on a grid we compute these

position	-4	-3	-2	-1	0	1	2	3	4
f'	1/280	-4/105	1/5	-4/5	0	4/5	-1/5	4/105	-1/280
f''	-1/560	8/315	-1/5	8/5	-205/72	8/5	-1/5	8/315	-1/560

Supplementary Table 2: Coefficients for central differences at 8th order.

quantities with a central finite difference using the appropriate coefficients up 8th order of accuracy. The parameters are reported in Table 2. Since the phases are stored in a matrix with the two dimensions representing the two lattice directions, the finite-size derivatives are computed along these vectors, so a coordinate transformation is needed to compute derivatives and Laplacians.

The initial condition of the minimization is taken to be a constant phase in the two layers. We consider many initial conditions with different phases to avoid getting stuck in local minima. At each step, we compute the functional derivative of the Hamiltonian $d\mathscr{H}$ (see Equation 6) and evaluate the energy of the new state $E(\varphi_1 + \eta d\mathscr{H}_1, \varphi_2 + \eta d\mathscr{H}_2)$ for a range of η and keep the one yielding the lowest energy. We update the phases and iterate this step until the energy converges to a stable value.

The finite-size nature of the derivatives makes it such that the shorter the distance between points in the grid, the better it approximates the continuum. In the results reported here, we use a 300×300 grid for biaxial strain and 500×3 for uniaxial strain in the a_1 direction.

4 Numerical results

We considered two types of strain in this work, biaxial strain (which corresponds to the case of two superimposed honeycomb lattices with different lattice lengths) and uniaxial strain along the a_1 direction (which yields an effective 1-D moiré unit cell). We expect the features to be stable also for more general cases. This can be understood through an easy argument. When straining or rotating one lattice with respect to the other, we are creating a moiré pattern. This pattern always starts from an AA-kind of stacking and has to end with another AA-stacking. The relative displacement between the two lattices in between always follows the same pattern. This means that, in the end, the main differences for what regards the interlayer interaction are the relative orientation of the two moiré vectors and their length. This also implies that the actual mechanism leading to the moiré pattern is not relevant in our calculation, it could also be a combination of strain (uniaxial, biaxial, shear ecc..) and relative rotation of the two layers.

The physical parameters entering in the Hamiltonian of our model are the intra-layer spin stiffness ρ_1, anisotropy d and the strain ϵ determining the moiré size (and the translation parameter t for the uniaxial case).

We find that for physically relevant parameters within an order of magnitude from the values presented in Table 1, the initial state (without an applied magnetic field) is a *tw-a* configuration. This happens in both the AA and the M' antiferromagnetic regions. In Figure 6a, we report a snapshot of the spin configuration in the two layers. When introducing and increasing the magnetic field, the AA region is the first to undergo a transition and align with the field (see Figure 6b). Subsequently, at higher fields, also the region near the M stacking undergoes a transition from a twisted-a to a twisted-s state (flop transition), as shown in Figure 6c.

We find that the features related to the spin flip-flop of the AA and M' regions are very stable to the choice of physical parameters. We show this by exploring a large window of parameters for the stiffness, anisotropy and strain. For the stiffness we consider the values $\rho = 0.1, 1.4, 5, 10, 100\,\text{meV}$, for the anisotropy $d = 0.01, 0.03, 0.0709, 0.11, 0.15\,\text{meV}$ and finally, for the strain we consider $\epsilon = 5\%, 4\%, 3\%, 2\%, 1\%, 0.5\%$. The magnetization curves as a function of the applied magnetic field show particular features when an antiferromagnetic region undergoes a transition. We can see a family of such curves in Figure 6 for biaxial (left) and uniaxial (right) strain. We can clearly see the features occurring at the flop transition of the M' r egion. The feature at the AA region is less evident in the biaxial strain than in the uniaxial case, because the area occupied by this antiferromagnetic region is much less in the former case.

The choice of physical parameters determines the position in the magnetic field at which the mentioned features appear. We went on to extract the main features of the magnetization plots in order to extract the tendencies upon variation of the parameters of the Hamiltonian. We computed magnetization plots for a broad range of parameters and we extracted the fields at which features appear. The results are shown in Supplementary Figures 11, 12, 13, and 14.

We can, first of all, appreciate how the difference in moiré periodicity becomes more and more important when increasing the spin stiffness. In particular, features happen at lower fields for smaller moiré unit cells. This occurs because there is a smaller region that needs to be flipped in order for the transition to happen.

From Supplementary Figures 12 and 14, we can see that the dependence of the transition field at AA is less dependent on the anisotropy than the one at M'. In general, the transition field decreases with the stiffness and the anisotropy. Also the strain plays an important role, with features happening at higher fields for smaller strain parameters, corresponding to big moiré unit cells.

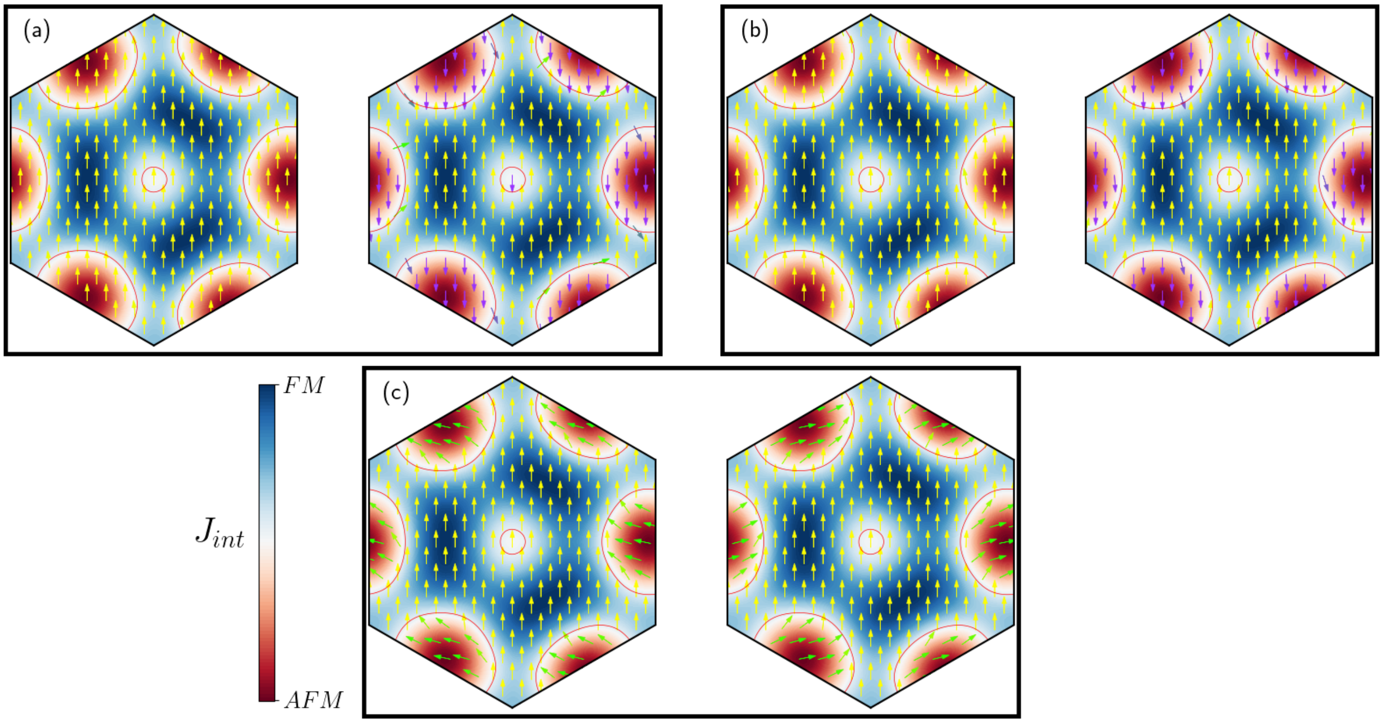

Supplementary Figure 9: **Spin texture in the two layers of our model for three prototypical cases.** In **(a)** a typical *tw-s* phase, where all AFM regions are pointing in the z direction. In **(b)** a configuration after the spin-flip of the region AA. We can start to see here also the boundary between the FM and AFM regions close to the M stacking. Finally, in **(c)** we report the spin texture of a typical configuration after the spin-flop transition of the M' region.

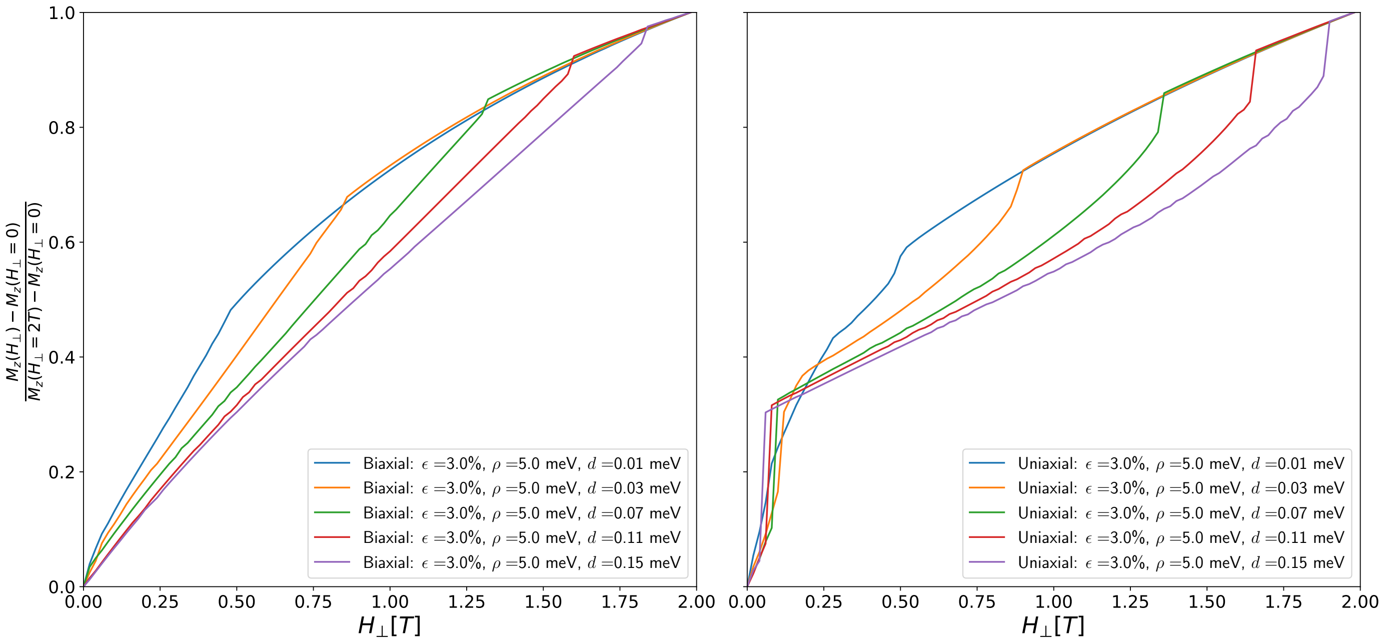

Supplementary Figure 10: Magnetization curves as a function of magnetic field for biaxial (left panel) and uniaxial (right panel) strain. The curves are at a fixed strain (3%) and stifness (5 meV), with varying anisotropy. Note that compared to the main text Fig. 6, we are considering here a wider range of parameters, including the applied magnetic field up to 2 Tesla.

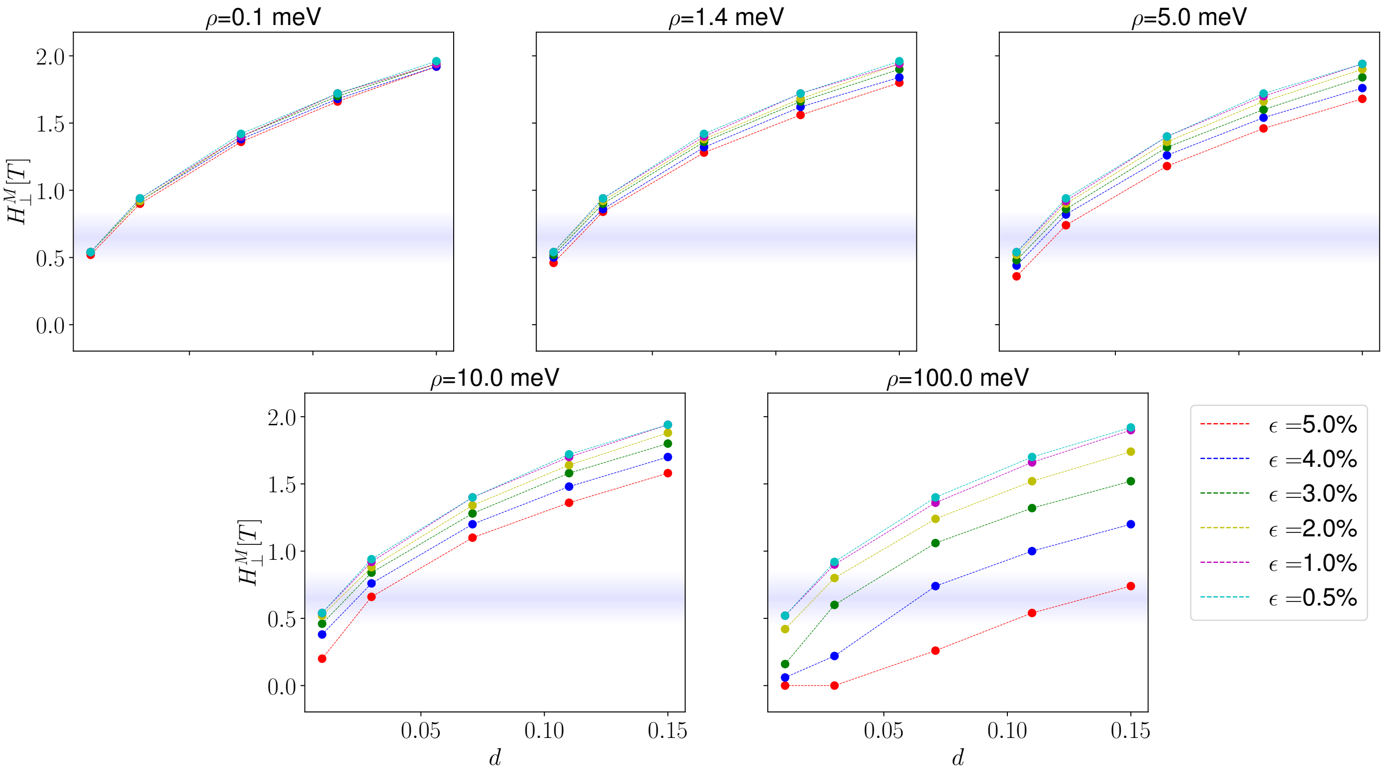

Supplementary Figure 11: Transition field of M’ region for different values of biaxial strain as a function of anisotropy. The blue shaded region refers to the range of applied magnetic fields where the spin flop transition in the M’ region occurs in the experimental samples.

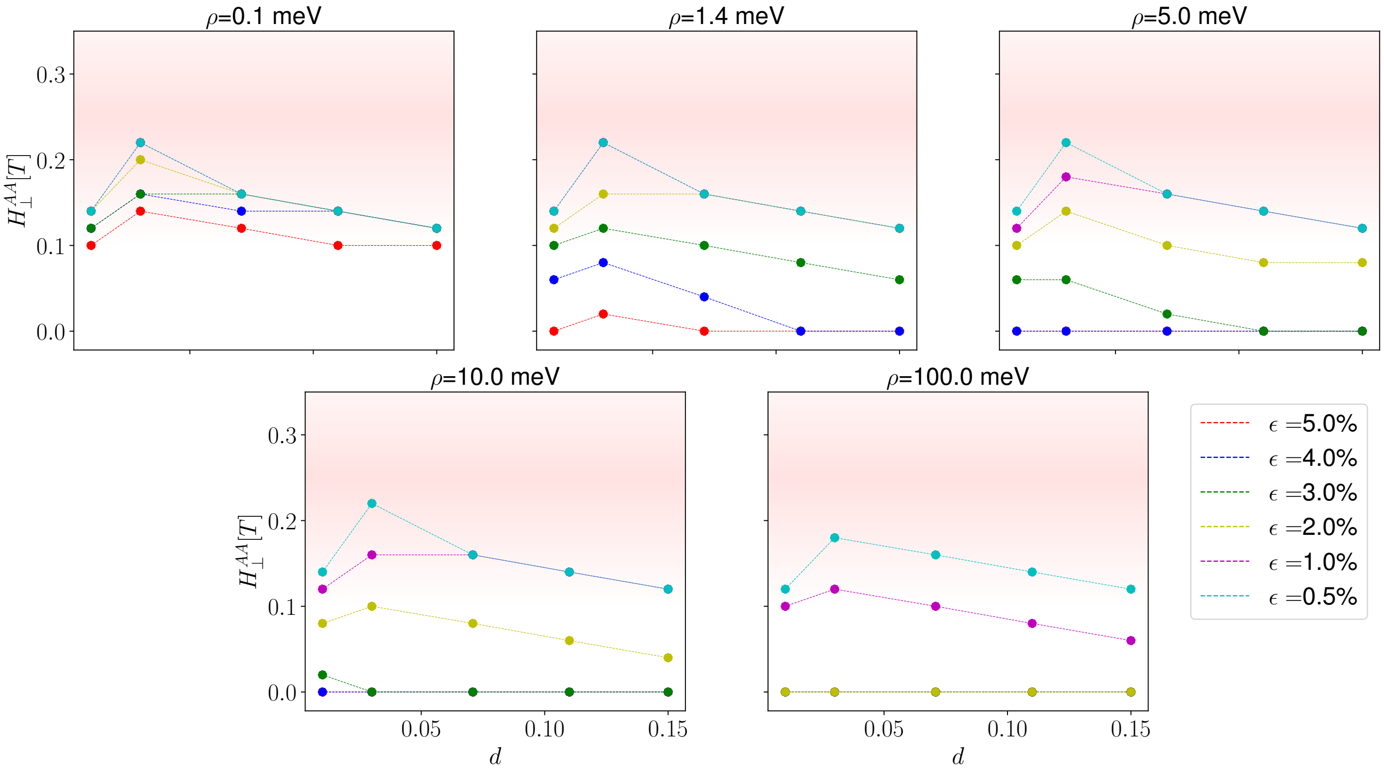

Supplementary Figure 12: Transition field of AA region for different values of biaxial strain as a function of anisotropy. The red shaded region refers to the range of applied magnetic fields where the spin flip transition in the AA region occurs in the experimental samples.

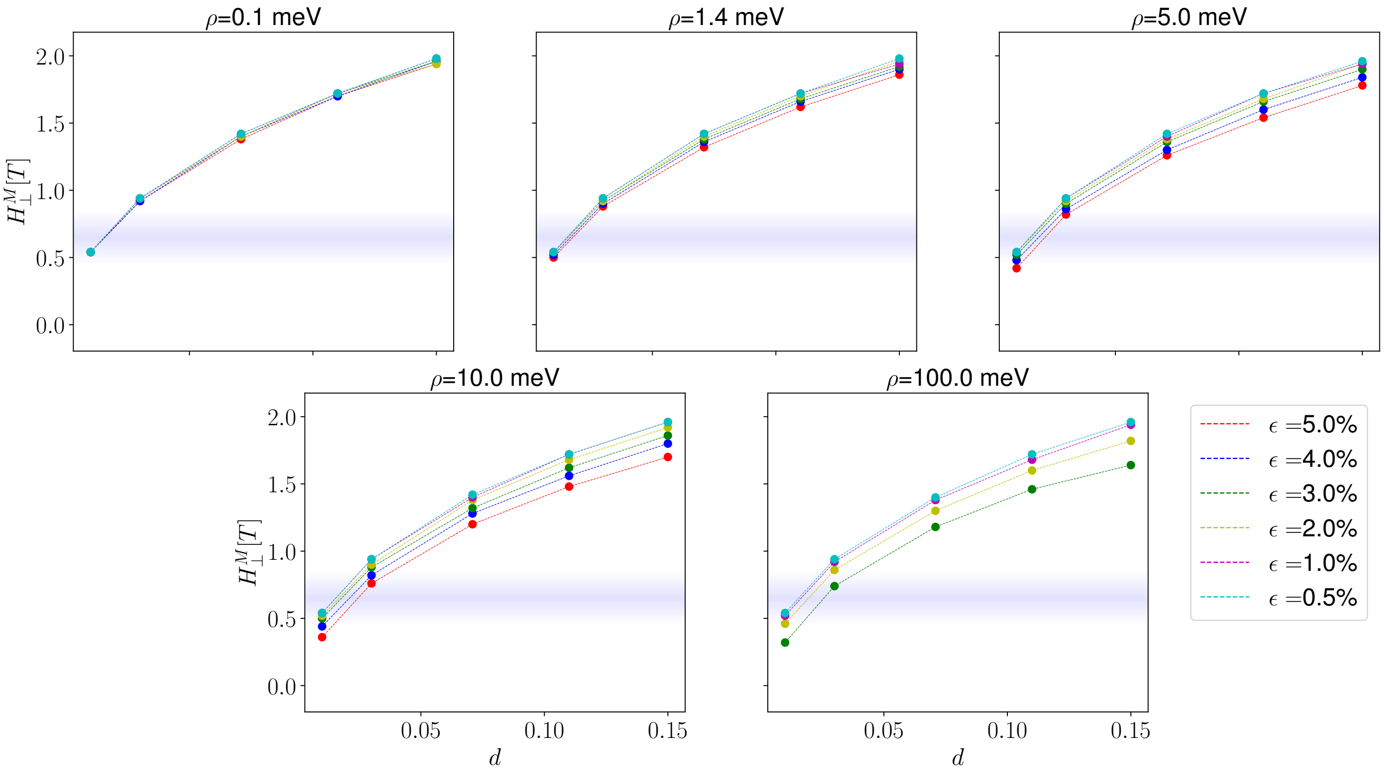

Supplementary Figure 13: Transition field of M' region for different values of uniaxial strain as a function of anisotropy. Here we considered no translation in the a_2 direction. The blue shaded region refers to the range of applied magnetic fields where the spin flop transition in the M' region occurs in the experimental samples.

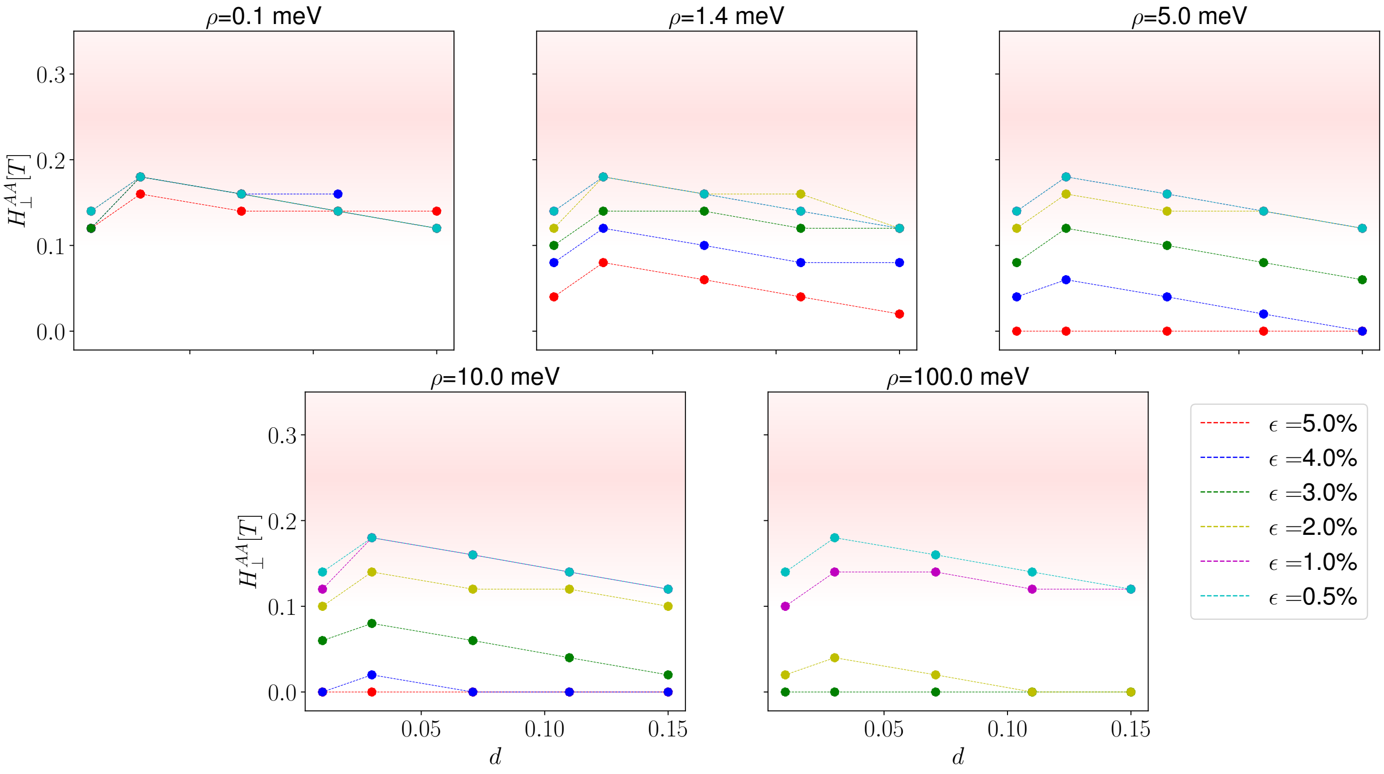

Supplementary Figure 14: Transition field of AA region for different values of uniaxial strain as a function of anisotropy. Here we considered no translation in the a_2 direction. The red shaded region refers to the range of applied magnetic fields where the spin flip transition in the AA region occurs in the experimental samples.

References

1 Akram, Muhammad et al. "Moiré Skyrmions and Chiral Magnetic Phases in Twisted CrX_3 (X = I, Br, and Cl) Bilayers". In: *Nano Letters* 21.15 (Aug. 2021). Publisher: American Chemical Society, pp. 6633–6639. ISSN: 1530-6984. DOI: 10.1021/acs.nanolett.1c02096. URL: https://doi.org/10.1021/acs.nanolett.1c02096 (visited on 10/27/2023).

2 Cai, Zhengwei et al. "Topological magnon insulator spin excitations in the two-dimensional ferromagnet $CrBr_3$". In: *Physical Review B* 104.2 (July 2021). Publisher: American Physical Society, p. L020402. DOI: 10.1103/PhysRevB.104.L020402. URL: https://link.aps.org/doi/10.1103/PhysRevB.104.L020402 (visited on 11/20/2023).

3 Fumega, Adolfo O. et al. "Electronic structure and magnetic exchange interactions of Cr-based van der Waals ferromagnets. A comparative study between $CrBr_3$ and $Cr_2Ge_2Te_6$". In: *Journal of Materials Chemistry C* 8.39 (2020), pp. 13582–13589.

4 Gibertini, Marco. "Magnetism and stability of all primitive stacking patterns in bilayer chromium trihalides". en. In: *Journal of Physics D: Applied Physics* 54.6 (Nov. 2020). Publisher: IOP Publishing, p. 064002. ISSN: 0022-3727. DOI: 10.1088/1361-6463/abc2f4. URL: https://dx.doi.org/10.1088/1361-6463/abc2f4 (visited on 10/27/2023).

5 Hejazi, Kasra, Luo, Zhu-Xi, and Balents, Leon. "Noncollinear phases in moiré magnets". In: *Proceedings of the National Academy of Sciences* 117.20 (May 2020). Publisher: Proceedings of the National Academy of Sciences, pp. 10721–10726. DOI: 10.1073/pnas.2000347117. URL: https://www.pnas.org/doi/full/10.1073/pnas.2000347117 (visited on 10/27/2023).

6 Nikitin, Stanislav E. et al. "Thermal evolution of Dirac magnons in the honeycomb ferromagnet $CrBr_3$". In: *Physical review letters* 129.12 (2022), p. 127201.

7 Owerre, S. A. "Chirality-induced magnon transport in AA-stacked bilayer honeycomb chiral magnets". en. In: *Journal of Physics: Condensed Matter* 28.47 (Sept. 2016). Publisher: IOP Publishing, 47LT02. ISSN: 0953-8984. DOI: 10.1088/0953-8984/28/47/47LT02. URL: https://dx.doi.org/10.1088/0953-8984/28/47/47LT02 (visited on 11/20/2023).

8 Owerre, S. A. "Magnon Hall effect in AB-stacked bilayer honeycomb quantum magnets". In: *Physical Review B* 94.9 (2016), p. 094405.

9 Owerre, S. A. "Topological honeycomb magnon Hall effect: A calculation of thermal Hall conductivity of magnetic spin excitations". In: *Journal of Applied Physics* 120.4 (2016).

10 Singh, Chandan K. and Kabir, Mukul. "Room-temperature ferromagnetism in two-dimensional $CrBr_3$". In: *Physical Review Materials* 6.8 (2022), p. 084407.

11 Sun, Wei et al. "Theoretical Investigation of Topological Magnetic Textures in Sliding Ferroelectric CrX_3 (X = Cl, Br, I) Moiré Superlattices: A Multiferroic Material with Unique Magnetoelectric Coupling for Information Storage Applications". In: *ACS Applied Nano Materials* 6.18 (Sept. 2023). Publisher: American Chemical Society, pp. 17021–17030. DOI: 10.1021/acsanm.3c03153. URL: https://doi.org/10.1021/acsanm.3c03153 (visited on 10/27/2023).

12 Tong, Qingjun et al. "Skyrmions in the Moiré of van der Waals 2D Magnets". In: *Nano letters* 18.11 (2018), pp. 7194–7199.

13 Wang, Zhe et al. "Magnetization dependent tunneling conductance of ferromagnetic barriers". In: *Nature communications* 12.1 (2021), p. 6659.

14 Yao, Fengrui et al. "Multiple antiferromagnetic phases and magnetic anisotropy in exfoliated $CrBr_3$ multilayers". en. In: *Nature Communications* 14.1 (Aug. 2023). Number: 1 Publisher: Nature Publishing Group, p. 4969. ISSN: 2041-1723. DOI: 10.1038/s41467-023-40723-x. URL: https://www.nature.com/articles/s41467-023-40723-x (visited on 10/27/2023).
